# Left ventricular restoration devices post myocardial infarction

**DOI:** 10.1007/s10741-018-9711-2

**Published:** 2018-05-17

**Authors:** Tom Hendriks, Remco A. J. Schurer, Lawien Al Ali, Ad F. M. van den Heuvel, Pim van der Harst

**Affiliations:** University of Groningen, University Medical Center Groningen, Department of Cardiology, 9700RB Groningen, The Netherlands

**Keywords:** Myocardial infarction, Left ventricular restoration, Transcatheter devices, Left ventricular remodeling

## Abstract

Even in the era of percutaneous reperfusion therapy, left ventricular (LV) remodeling after myocardial infarction (MI) leading to heart failure remains a major health concern. Contractile dysfunction of the infarcted myocardium results in an increased pressure load, leading to maladaptive reshaping of the LV. Several percutaneous transcatheter procedures have been developed to deliver devices that restore LV shape and function. The purposes of this review are to discuss the spectrum of transcatheter devices that are available or in development for attenuation of adverse LV remodeling and to critically examine the available evidence for improvement of functional status and cardiovascular outcomes.

## Introduction

Despite continuous improvements in acute and long-term treatment of myocardial infarction (MI) over recent decades, the resulting myocardial injury remains an important cause of adverse left ventricular (LV) remodeling, which over time progresses into the clinical syndrome of heart failure [1]. Typical for post-MI LV remodeling is dilatation, geometric change (from ellipsoid to spherical), and scar formation [2]. Both functional and structural measures of adverse LV remodeling, such as LV ejection fraction (LVEF) and LV dimensions, are associated with mortality and cardiovascular events after MI [3–6]. The beneficial effects of drugs or medical devices on LV remodeling measures have been associated with reduced long-term mortality [7]. In this review, we will focus on the role of percutaneous transcatheter delivered medical devices on LV remodeling. The definition of “medical device” varies according to local regulations, but generally, it is defined as an instrument, apparatus, software, or material that is intended for use in the diagnosis, treatment, monitoring, or prevention of disease [8]. It can be differentiated from a drug by its mechanism of action, which is not pharmacological, immunological, or metabolic. Numerous devices have been developed that attempt to restore the LV shape and revert adverse post-MI changes, intended to provide long-term benefits in terms of survival and functional status. Here, we will provide a critical overview of the empirical evidence supporting the use of LV restoration devices at various stages of LV remodeling post-MI, with a main focus on the transcatheter delivery route.

## Remodeling after myocardial infarction

In the early stages after MI, influx of inflammatory cells leads to the destruction of collagen and regional thinning of the infarcted area [9]. The healing of necrotic tissue and formation of granulation tissue involves interactions among inflammatory cells such as macrophages, fibroblasts, and myofibroblasts [10]. As the infarcted region expands, cardiac fibroblasts deposit collagen in the infarct zone, which leads to scar formation and prevents further dilatation. Typical for post-MI remodeling is an increase in LV volumes and a more spherical LV shape due to a loss of myocytes and stretching of the remaining myocytes by the addition of sarcomeres in series [11]. Theoretically, dilatation can be beneficial by maintaining stroke volume through the Frank-Starling mechanism. However, the LV radius and the inverse of wall thickness are proportional with wall stress (law of Laplace). Consequently, LV dilatation increases wall stress and extends the burden on the remaining myocytes, leading to subendocardial myocardial ischemia and ultimately causing more damage [12]. Moreover, impaired LV contractility and reduced cardiac output after MI leads to the activation of neurohormonal pathways [13]. These pathways are thought to act as a way to maintain cardiac output through inotropic and chronotropic effects. However, these compensatory mechanisms result in an increased workload for the remaining myocytes, leading to progressive adverse remodeling. Drug therapies aimed at preventing heart failure post-MI mostly target neurohormonal pathways. Numerous studies have found evidence of a favorable effect of these drugs on parameters of LV remodeling and patient outcome [2]. Progressive dilatation is associated with larger infarct mass, nonscarred LV mass, and ongoing ischemia [14, 15]. It can lead to further hemodynamic consequences such as functional mitral regurgitation (FMR), which occurs in 20–25% of patients after MI and is associated with higher mortality rates [16]. Patients with progressive LV dilatation will eventually develop symptoms of heart failure. Despite very effective medical treatment options, the current chance of developing heart failure within 5 years after MI is 17% in men and 21% in women above the age of 45 [17], which occurs most distinctly in patients admitted with an anterior MI [18].

## Guideline recommendations

Signs of LV remodeling can already be seen in the early stages after MI, within hours to days [2]. Contemporary guidelines recommend the use of transthoracic echocardiography (TTE) within the first 3 days after MI, to assess LVEF [19, 20]. A repeat TTE assessment 30–90 days after initial hospitalization is recommended because cardiac function can still recover in the case of myocardial stunning and hibernation. Cardiac magnetic resonance imaging (CMR) and computed tomography imaging (CT) are more suitable imaging modalities for research as a smaller sample size is needed to detect changes in LV structure, due to higher accuracy and reproducibility [21]. Recommended long-term therapies post-MI are based on cardiac rehabilitation, lifestyle interventions, and medical therapy. Contemporary MI guidelines do not recommend the use of transcatheter devices in long-term therapies post-MI [19, 20]. In the most recent heart failure guidelines [22–24], implantable cardioverter-defibrillators (ICDs), defibrillators with cardiac resynchronization therapy (CRT-Ds), and cardiac contractility modulation (CCM) are the only transcatheter devices recommended in the treatment of heart failure. This highlights that LV restoration devices have not yet been accepted as standard clinical practice.

## Devices to reverse left ventricular remodeling

Short-term effects of drugs and devices on (reverse) LV remodeling are associated with reduced longer-term mortality rates [7]. Consequently, LV remodeling parameters are often used as alternatives or proxy variables for long-term mortality in clinical trials, because a smaller number of patients and a shorter follow-up are required to attain the same power. In the 1950s, the strong relation between adverse LV remodeling and patient outcome led to the hypothesis that surgical restoration of the original volume and ellipsoid shape of the LV could be beneficial in cases of severe LV remodeling. In the following paragraphs, we will discuss several surgical and transcatheter devices (Table 1) that have been investigated in post-MI patients in attempts to restore the LV shape as well as its hemodynamic and mechanical properties. The order of discussion is based on the indication for use.

### Dilated ischemic cardiomyopathy

The first surgical attempt at LV restoration was the aneurysmectomy with a linear suture, first described by Cooley et al. in 1958 [25] and developed over the years [26]. The procedure was used in a selected group of patients with previous anterior MI and an aneurysmatic LV. The Dor procedure, using a circular suture and a pericardial patch that was covered by the residual myocardium, was later deemed superior [27]. In the STICH randomized controlled trial (*N* = 1000), CABG and surgical ventricular reconstruction reduced LV end-systolic volume by 19%, compared with 6% by CABG alone, but had no significant effect on mortality, hospitalization for cardiac events, and 6-min walk test (6MWT) distance [28]. A possible reason for this outcome proposed by the authors is that surgical reduction of LV volume, in addition to reducing wall stress, also reduces diastolic distensibility.

Several surgical LV restoration techniques have been investigated in patients with dilated cardiomyopathy, including not only ischemic but also idiopathic etiologies. In 1985, the first successful dynamic cardiomyoplasty procedure was performed, which was the surgical wrapping of an autologous latissimus dorsi muscle around the heart, which was activated by an external cardiomyostimulator for 10 weeks to gradually transform muscle fibers from type II to type I [29]. A prospective study including 68 patients observed a small increase in LVEF at 6 months (*p* = 0.05), but no significant change in peak oxygen consumption or cardiac index [30]. The cardiomyoplasty-skeletal muscle assist randomized trial (C-SMART) aimed to randomize 400 patients, but was terminated prematurely due to problems with patient recruitment and reimbursement. As opposed to autologous tissue, the LV can also be wrapped with synthetic material. Ventricular restraint therapy is the surgical placement of a multifiber polyester mesh around the LV, designed for patients with a dilated cardiomyopathy. It is intended to restore the ellipsoid shape of the LV and alleviate wall stress. In the ACORN trial (*N* = 300), the implanted device named the CorCap™ (formerly Acorn Cardiovascular, St. Paul, MN, USA) had no significant effect on 3- and 5-year mortality but did significantly reduce LV end-diastolic volume (LVEDV) up to 5 years after implantation, consistent across strata with and without mitral valve replacement [31–34]. It has to be noted that these results cannot be extrapolated to post-MI patients, since only 10% of the included patients had heart failure with an ischemic etiology.

#### Epicardial ventricular restoration

As an alternative to the invasive surgical ventricular reconstruction method, a minimally invasive surgical technique was designed to exclude the nonviable part of the LV in patients with LV dilatation and anteroseptal scarring. The Revivent™ myocardial anchoring system (BioVentrix, San Ramon, CA, USA), previously named the PliCath HF™, is composed of polyester-covered titanium anchors (5 × 25 mm) mounted on a polyethylene-ether-ether-ketone tether, which are placed on the right side of the interventricular septum and on the LV wall. The anchors are drawn together to allow apposition of the LV free wall to the septum, thereby excluding the nonviable anteroseptal scar. Initial results in humans (*N* = 11) demonstrate a stable reduction in LV volumes up to 12 months [35]. The Revivent system has received CE marking for commercial use in Europe.

Later developments of the device led to the introduction of a transcatheter component and a minor name change to Revivent-TC™ (transcatheter) system, also receiving CE marking. The upgraded system is not completely transcatheter-based, still requiring a less invasive left thoracotomy. From outside the LV, a needle is used to puncture the LV wall, cross the LV, and puncture the interventricular septum. The needle position is monitored using fluoroscopy, and a Swan-Ganz catheter is introduced in the right jugular vein to monitor ventricular pressures. After reaching the right ventricle, the needle is replaced with a sheath and a guidewire. The guidewire is captured in the right ventricle by a snare from the Swan-Ganz catheter and connected with the internal anchor. The external anchor is placed from outside the LV wall. If necessary, additional pairs of anchors (2–3) are placed to achieve the desired line of apposition. Injection of contrast in the LV is used to confirm whether successful exclusion of the nonviable LV segment has been achieved. The Revivent-TC system has been successfully implanted in six sheep, successfully reducing LVESV, improving LVEF, and improving strain in border and infarct regions [36]. Study results from 51 patients treated with the Revivent and 20 patients treated with the Revivent-TC have been presented in the form of an abstract but have not yet been published in a peer-reviewed journal [37]. A prospective, multicenter, dual-arm pivotal study is currently aiming to include 146 subjects, who will be randomized to the Revivent-TC system or optimal medical therapy in a 2:1 ratio. Major inclusion criteria are the presence of an acontractile scar in the septal and anterior, apical or anterolateral regions of the LV, viable myocardium in the remote regions, LVEF under 45%, LVESVi larger than 50 ml/m^2^, and NYHA class II or higher.

#### Transcatheter ventricular partitioning

The umbrella-like Parachute® device (Cardiokinetix, Redwood City, CA, USA) is intended to partition off the akinetic or aneurysmatic portion of the LV in patients with ischemic heart failure. The device is comprised of a fluoropolymer (ePTFE) membrane stretched over a self-expanding nitinol frame, ranging between 65 and 95 mm in diameter when expanded. It is deployed into the LV apex and stabilized by 2-mm anchors at the end of each strut of the umbrella. The device provides efficacy by regional unloading of the akinetic LV region and global reduction of wall stress by reducing LV dimensions. Before implantation, LV anatomy has to be evaluated carefully, preferably by computed tomography imaging (CT), because anatomical characteristics such as prominent trabeculation or a “LV moderator band” are unsuitable for device implantation. The Parachute device has received CE marking for commercial use in Europe but is only approved for investigational use in the USA.

The device has been investigated in several PARACHUTE trials (Table 1). The PARACHUTE First-In-Human trial (*N* = 39), which took place in Europe and the USA simultaneously, demonstrated safety and feasibility of the Parachute device in heart failure patients with LVEF between 15 and 40% and a dilated LV with an akinetic or dyskinetic anterior-apical wall [38]. There was a stable and significant reduction in LVEDV up to 3 years after implantation [39]. However, stroke volume and LVEF were also significantly lower at 3 years compared with measurements at baseline. The PARACHUTE III trial was a prospective, nonrandomized observational study conducted in Europe, including 100 subjects with similar inclusion criteria to the First-In-Human trial [40]. Procedural success was achieved in 97% of the subjects. LV volumes were significantly reduced at 12 months, and mean 6MWT distance improved from 372 m at baseline to 397 m (*p* < 0.01). During 12-month follow-up, cardiac mortality was 8.4%, device-related major adverse cardiac cerebral events (MACCE) occurred in 7.0%, heart failure hospitalizations in 24.1%, and stroke in 3% of subjects. Even though patients were given low-dose aspirin and warfarin for at least 12 months post device implant, the echo core lab identified thrombus on the device in 3.3% of the subjects at 12 months. The PARACHUTE IV was the first randomized controlled trial, aiming to include 478 patients with NYHA class III–IV, LVEF between 15 and 35%, LV motion abnormalities, and LV anatomy appropriate for Parachute implantation assessed by CT imaging and randomize to optimal medical therapy or Parachute implantation [41]. The primary endpoint was death or hospitalization for worsening heart failure. After including 331 subjects, the PARACHUTE IV trial was terminated in June 2017 (clinicaltrials.gov≠NCT01614652) and it is unclear whether investigation of the device will be continued.

In addition to the primary study results, there have been some interesting post hoc analyses. Hemodynamic assessment after Parachute implantation demonstrated an acute increase in stroke volume and mean aortic pressure [42]. A CT study investigating diastolic parameters before and after Parachute implantation observed that the Parachute device significantly reduced diastolic parameters such as the ratio between transmitral velocity in early diastole and early diastolic mitral septal tissue velocity (*E*/*E*′), and this reduction was correlated with improvement in NYHA functional class [43]. Another CT study observed a favorable effect of the Parachute on mitral valve geometry, by reducing interpapillary muscle distance and tenting height [44]. A pathology study investigated seven Parachute devices after cardiac transplantation (*N* = 3) and at autopsy after deaths that were not device related (*N* = 4) [45]. The devices generally showed good healing by organized endocardial tissue growth on luminal and abluminal surfaces of the membrane. Parachute devices of long duration (> 2 years, *N* = 2) showed evidence of microscopic calcification, which could in theory lead to fatigue and rupture of the membrane on the long term.

The aforementioned study findings suggest that the Parachute device could be beneficial by reducing cardiac dimensions and end-diastolic wall stress and improving cardiac output. However, 3-year results demonstrate a reduction of LVEF and stroke volume. Results from the PARACHUTE IV trial will have to demonstrate the added benefit of the Parachute device in addition to optimal medical therapy. It is unclear whether the investigation and development of the device will be continued.

### Ischemic mitral regurgitation

Several surgical LV techniques have been attempted to treat ischemic FMR before the use of a transcatheter delivery route. The Coapsys™ (formerly Myocor, Maple Grove, MN, USA) device was designed to treat annular dilatation and papillary muscle displacement in patients with FMR by connecting an anterior and posterior epicardial pad with a subvalvular chord through the LV cavity and subsequently reducing the anteroposterior diameter by bearing load during the diastolic phase. The RESTOR-MV randomized controlled study was initiated, including patients referred for CABG with the presence of FMR [46]. Participants were stratified according to the need for mitral valve repair, as assessed by the surgeon. They were subsequently randomized to undergo either CABG (± mitral repair) or CABG and Coapsys implantation. The study was unfortunately terminated early due to insufficient funding. The investigators did find a significant survival advantage for patients who received the Coapsys device in the 165 subjects that were included, even though controls showed lower FMR grades during follow-up. In 2008, Edwards Lifesciences (Irvine, CA, USA) purchased the rights for the Coapsys device but since then has not revealed any plans for continuing the investigation or further development of the device.

#### Transventricular system

A transcatheter variant of the Coapsys, the iCoapsys™ repair system, was designed for the same patient category with ischemic FMR. The device consisted of the same elements as the original Coapsys (epicardial pads and a transventricular chord) but was implanted using transcatheter delivery. It was successfully implanted in 12 adult sheep, with no complications reported [47]. Prior to the start of a first-in-human study in 2008, Edwards Lifesciences purchased the rights for the iCoapsys device and discontinued the trial.

Similar to the iCoapsys repair system, the percutaneous septal sinus shortening system or PS^3^ System™ has a bridge element inside the LV cavity, but instead of being attached on the epicardium, its anchors are placed on the right side of the atrial septum and in the great cardiac vein. It has been investigated in an ovine tachycardia model (*N* = 19), successfully improving cardiac output and reducing FMR grade and septolateral diameter [48]. First-in-human implantations in two patients, immediately prior to explantation during planned clinically indicated surgical mitral valve repair, reduced septal-lateral dimensions and FMR grade [49].

#### Transvenous and subannular annuloplasty

Transcatheter interventions targeting the mitral apparatus such as percutaneous edge-to-edge mitral valve repair, percutaneous annuloplasty, and percutaneous mitral valve replacement are being reviewed elsewhere in this issue. A retrospective analysis of percutaneous edge-to-edge repair with the MitraClip (Abbott Vascular, Menlo Park, CA, USA) in 106 patients with ischemic FMR showed a significant reduction of left atrial volume at 1 year after implantation but no effect on LV volumes [50]. In the EVEREST II trial, MitraClip was associated with durable reduction of LVEDV up to 5 years, but this trial included mostly patients with degenerative mitral regurgitation [51]. In the following paragraph, we will discuss several transcatheter techniques here that target the coronary sinus system and the subannular space to reshape the LV with an effect on the mitral apparatus (Table 1).

A transcatheter mitral annuloplasty by implantation of a device in the coronary sinus (transvenous annuloplasty) has the advantage that no atrial septum puncture is required to gain access to the left heart. However, the coronary sinus is anatomically at a distance from the mitral annulus, and in some cases, the great cardiac vein passes over a coronary artery, which can be compressed or occluded by the device. The Monarc (previously Viking) system (Edwards Lifesciences, Irving, CA, USA) uses an implant with two self-expanding anchors and a spring-like “bridge,” which was investigated in 72 patients of which 57% had a prior MI [52]. It was implanted in 59 patients (82%), and the primary safety endpoint of freedom from death, tamponade, or MI was 91% at 30 days and 82% at 12 months. A major issue in patients with implanted Monarc devices was coronary artery compression, which was observed in 15 patients who underwent follow-up angiography (30%), of which two presented with an acute MI. Another alternative, the percutaneous transvenous mitral annuloplasty (PTMA) device (formerly Viacor, Wilmington, MA, USA) consists of a PTMA catheter and nitinol rods of varying stiffness and is intended for patients with moderate to severe FMR, NYHA class II–IV, and LVEF 20–50%. The PTOLEMY-2 safety and feasibility trial included 43 subjects in whom implantation was attempted, resulting in 30 successful implantations of the device [53]. Unfortunately, four devices had to be removed at a later stage and two periprocedural deaths led to early termination of the trial by the sponsor. Also, during long-term follow-up after implantation, late erosions of the coronary sinus led to serious complications, in some cases fatal [54, 55]. The most thoroughly investigated transvenous annuloplasty device is the Carillon Mitral Contour System (Cardiac Dimensions, Sydney, Australia), which uses an implant that is composed of two self-expanding nitinol (nickel-titanium alloy) anchors connected by a curvilinear segment and delivered by a catheter via the right internal jugular vein, previously described in more detail [56, 57]. There have been three nonrandomized safety and efficacy studies conducted in Europe, including patients with at least moderate FMR, LVEF under 40%, NYHA functional class II or higher, and 6MWT distance between 150 and 450 m [56–58]. Where reported, FMR etiology was ischemic in 55–60% of patients. Study results demonstrated consistent low 30-day major adverse event rate (between 2 and 13%), significant reductions of regurgitant volume, and improvements in 6MWT distance. During follow-up evaluation in the TITAN trial, nine subjects (25% of subjects with a permanent implanted device) were observed to have a fractured anchor wire [57]. Although this was not linked to the occurrence of clinically relevant adverse events, the device (XE2) was modified to help reduce strain in the proximal anchor. The modified device (mXE2) was evaluated in the TITAN II trial, resulting in no more fractured devices [58]. The REDUCE FMR multicenter randomized controlled double-blind trial is currently randomizing 180 patients to the Carillon Mitral Contour System or optimal medical therapy in a 3:1 ratio, with primary efficacy endpoint being improvement in regurgitant volume at 12 months, assessed by an independent echocardiography core laboratory blinded to patient data [59]. A more recent device, the mitral loop cerclage (Tau-PNU Medical, Pusan, Korea), consists of a tension element made of stainless steel and an arch-like coronary artery protection element. The device creates a loop through the coronary sinus and the right ventricle across the interventricular septum, covering the full circumference of the mitral annulus and applying circumferential tension. Because of the coronary artery protection device, it is suitable for a wider range of patients than the previously described devices. It has been investigated in a first-in-human feasibility study on five subjects with severe FMR and NYHA class III or IV, reporting a successful implantation in four subjects, reducing LVEDV and regurgitant volume [60]. Future studies will have to determine whether a transvenous mitral annuloplasty can be a feasible treatment for patients with ischemic FMR (Table 1).

**Table 1:** Left ventricular restoration devices used in various stages after myocardial infarction

	Type of device	Device	Indication*	Trials	Status	Primary endpoints	No. of subjects (nonischemic)
Early phase	Intracoronary infusion of biomaterial	Sodium alginate + calcium gluconate BCM	2–5 days after large STEMI and successful revascularization	First-in-human [61]	Completed	180-day symptomatic heart failure *(3.7%*), renal failure (*3.7%*), stroke death (*0%*)	27 (0)
				PRESERVATION 1 [62]	Completed	6-month ∆LVEDVi (*BCM 14.1 ± 28.9 ml/m*^*2*^ vs. *saline 11.7 ± 26.9 ml/m*^*2*^*; p = 0.49*)	306 (0)
	Intramyocardial injection of biomaterial	Calcium hydroxyapatite microspheres (CHAM)	Directly post-MI	Animal studies [63–65]	Completed	Infarct expansion, proteolytic pathways, LVEF, regional contractile strain, MR severity	–
		Injectable hyaluronic acid-based hydrogel	Directly post-MI	Animal studies [66, 67]	Completed	Global LV remodeling, infarct thinning and expansion, and infarct stiffness	–
		Myocardial matrix hydrogel (VentriGel)	60 days–3 years after STEMI, LVEF 25–45%	First-in-human (*NCT02305602*)	Recruiting	6-month SAE	18 (0)
Late phase	Ventricular partitioning	Parachute	LVEF < 40%, abnormal LV wall motion, suitable LV anatomy	PARACHUTE first-in-human [39]	Completed	Implantation success and 6-month freedom from device-related MACE (*74%*)	39 (0)
				PARACHUTE III [40]	Completed	1-year procedural- or device-related MACCE (*7%*)	100 (0)
				PARACHUTE IV [41]	Terminated	Death or rehospitalization for worsening heart failure	331 (0)
				PARACHUTE V (*NCT02543632*)	Terminated	Quality of life	85 (0)
	Epicardial ventricular restoration	Revivent-TC	LVEF < 45%, dilated LV, acontractile scar, NYHA class II–IV	ReviventTC1 (*NCT02553785*)	Recruiting	6-month SAE and ∆LVESVi	50 (0)
				Revivent-TC System Clinical Study (*NCT02931240*)	Recruiting	Effectiveness, compared to surgical treatment and medical therapy	126 (0)
	Transventricular system	iCoapsys	Severe FMR, NYHA class II–III	First-in-human (*NCT00512005*)	Terminated	Intra- and periprocedural safety and MR reduction	? (?)
		Percutaneous septal sinus shortening (PS^3^) system	FMR	Animal studies [48]	Completed	Septal-lateral dimensions (*− 17%*), mean force on bridge (*1.16–1.87 N*)	–
				First-in-human [49]	Completed	Septal-lateral dimensions (*− 29%*), MR grade reduction (*1 and 2*)	2 (1)
	Transvenous annuloplasty	Monarc	Moderate to severe FMR, dilated LV, LVEF > 25%	EVOLUTION [52]	Completed	30-day freedom from death, tamponade, and MI (*91%, 82% at 1 year*)	72 (23)
		Percutaneous transvenous mitral annuloplasty (PTMA)	Moderate to severe FMR, NYHA class II–IV, LVEF 20–50%	PTOLEMY-2 [53]	Terminated	30-day freedom from MACEs and 6-month MR reduction	43 (13–26)
		Carillon	Moderate to severe FMR, dilated LV, LVEF < 50%, 6MWT distance 150–450 m	AMADEUS [56]	Completed	30-day MAE (*13%*)	48 (?)
				TITAN [57]	Completed	30-day MAE (*1.9%*)	53 (19)
				TITAN II [58]	Completed	30-day MAE (*2.8%*)	43 (15)
				REDUCE FMR [59]	Active, not recruiting	1-year ∆ regurgitant volume	180 (?)
				CARILLON (*NCT03142152*)	Recruiting	1-year freedom from MAE, ∆ regurgitant volume, and clinical composite of death, time to first HF hospitalization and improvement in 6MWT distance	400 (?)
		Mitral loop cerclage	Severe FMR, NYHA class III–IV	First-in-human [60]	Completed	1-month freedom from MACE (*80%*), reduction of regurgitant volume (*66%*) and effective regurgitant orifice area (*76%*)	5 (2–4)
	Direct mitral valve annuloplasty (ventriculoplasty)	AccuCinch	Moderate to severe FMR, NYHA class II–IV, LVEF ≥ 20%	Safety and efficacy trials (*NCT00800046, NCT01899573, NCT02806570, NCT02153892, NCT03183895*)	Recruiting	30-day device-related or procedure-related MAE, MACE, acute and 30-day MR reduction	197 (?)

*6MWT*, 6-min walk test; *BCM*, bioabsorbable cardiac matrix; *FMR*, functional mitral regurgitation; *HF*, heart failure; *LV*, left ventricle; *LVEF*, left ventricular ejection fraction; *LVEDVi*, left ventricular end-diastolic volume indexed to body surface area; *LVESVi*, left ventricular end-systolic volume indexed to BSA; *MACCE*, major adverse cardiac cerebral events; *MACE*, major adverse cardiac events; *MAE*, major adverse events; *MI*, myocardial infarction; MR, mitral regurgitation; *No.*, number; *NYHA*, New York Heart Association; *SAE*, serious adverse events; *STEMI*, ST-elevation myocardial infarction

*Inclusion criteria occasionally vary in case of multiple trials. The indications mentioned below are generalized and not intended to reflect the full scope of in- and exclusion criteria

The AccuCinch (Ancora Heart, Santa Clara, CA, USA) transcatheter direct mitral valve annuloplasty (or ventriculoplasty) system is designed to place anchors in the subannular space [68]. Cinching of these anchors reduces both basal LV and mitral annular dimensions. Anchors are delivered by anchor delivery catheters that are introduced through a modular guide tunnel via the femoral artery. A safety and feasibility trial is currently conducted, aiming to include a total of 40 patients.

### Early phase after myocardial infarction

**Fig. 1:**
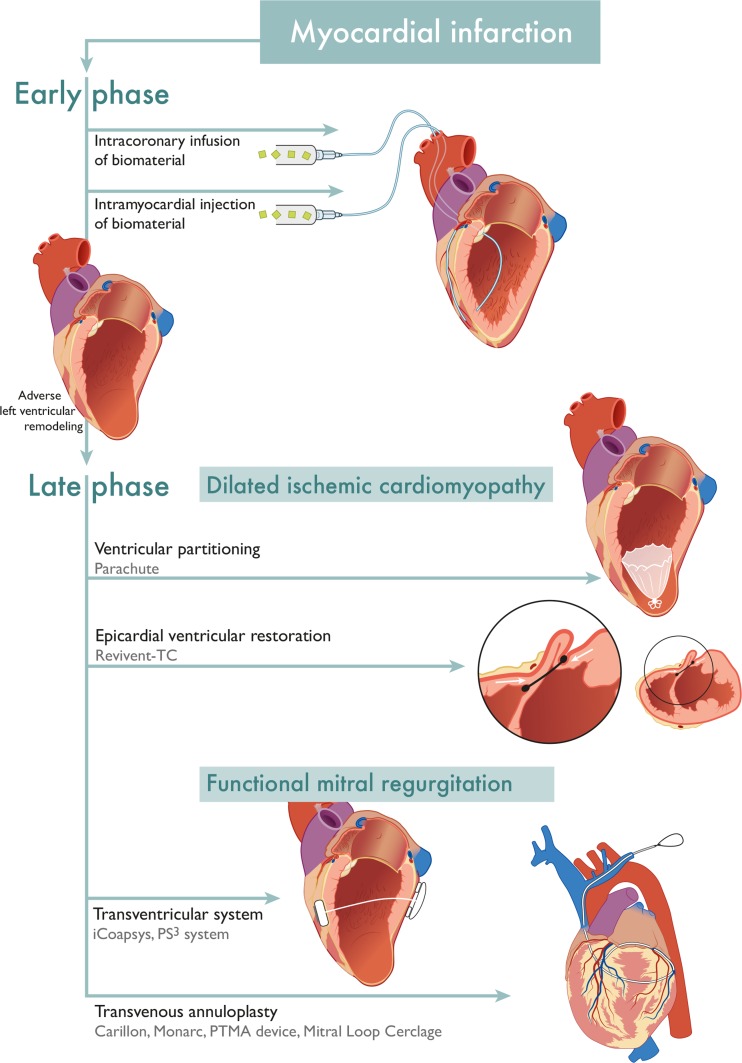
An overview of the discussed transcatheter left ventricular restoration devices, grouped by indication for use based on the stage of adverse left ventricular remodeling post myocardial infarction. The represented transvenous annuloplasty device is the mitral loop cerclage, the other devices are differently shaped

LV reconstruction techniques are intended to (partially) revert the changes to LV mechanical properties caused by myocardial injury. Ideally, this should be done during the early stages after MI, when scar formation, regional thinning of the myocardium, and LV dilatation can still be prevented. Most of the aforementioned studies, however, exclude patients within 90 days after MI. The reasoning behind this is that adverse LV remodeling can largely be reversed due to myocardial hibernation. Early assessment of myocardial viability by late enhancement CMR could be used to assess the risk of progressive LV remodeling and the need for an early intervention [69] (Fig. 1).

#### Biomaterial injection and infusion

As stated earlier, MI induces degradation of extracellular matrix, which reduces elasticity of the myocardium. A proposed option to restore the mechanical properties of the myocardium is by injecting biomaterials into the myocardium, which could serve as an artificial extracellular matrix. Biomaterial scaffolds incorporating stem cells were not included in this review, because we considered cellular products to be a different category. Several types of biomaterial injections have been tested in animal studies. The effect of calcium hydroxyapatite microsphere (CHAM) injections in infarcted myocardium was investigated in 31 sheep and appeared to limit LV dilatation and improve LVEF up to 4 weeks after ligation of the left anterior descending coronary artery [63]. A follow-up study randomized 24 adult male sheep to receive either saline or CHAM injection after ligation of the left anterior descending coronary artery [64]. In addition to improving LVEF and reducing LVEDV compared to controls, the authors observed a reduction of collagen content in the sheep that received CHAM injections. A more recent study randomized 25 Yorkshire swine to receive either 20 normal saline or CHAM injections at 30 min after ligation of the circumflex artery [65]. The animals receiving CHAM injections showed reduced infarct thinning and progressive improvement in border zone and global LV function. To date, no studies in humans have been reported.

Another widely investigated biomaterial is injectable myocardial matrix hydrogel, which is produced by decellularizing porcine myocardial tissue and processing it to form a myocardial matrix with the ability to gel upon injection. Myocardial matrix hydrogel injections have been investigated in 58 rats undergoing ischemia reperfusion followed by injection of either the hydrogel or saline 2 weeks later [70]. The hydrogel increased endogenous cardiomyocytes in the infarct area and maintained cardiac function without inducing arrhythmias. Similar results were observed in a study on 10 pigs, using transcatheter injections [71]. The authors also investigated the hemocompatibility of porcine myocardial matrix with human blood and observed no effect on clotting times. Possible mechanisms underlying the benefit of myocardial matrix injections are an altered inflammatory response, reduced cardiomyocyte apoptosis, enhanced neovascularization, diminished cardiac hypertrophy and fibrosis, and enhanced recruitment of progenitor cells [72]. A phase 1 study is currently enrolling post-MI patients to study the safety of transcatheter administration of myocardial matrix hydrogel in humans.

A less frequently investigated type of hydrogel that has been investigated in animal studies post-MI is injectable hyaluronic acid-based hydrogel, which appears to reduce myofiber stress and limit adverse LV remodeling [66, 67].

As a less invasive alternative to intramyocardial injections, biomaterials can also be administered by intracoronary infusion. In 27 post-MI patients, the intracoronary administration of 2 ml of 1% sodium alginate plus 0.3% calcium gluconate within 7 days after MI was feasible and tolerated well [61]. The infusion is assumed to permeate infarcted tissue, where it cross-links into a hydrogel and forms a bioabsorbable cardiac matrix (BCM). The PRESERVATION I randomized controlled trial randomized 303 patients at 2–5 days after ST-elevation MI (STEMI) with TIMI (Thrombolysis In Myocardial Infarction) flow grade 3 in the infarct-related coronary artery to intracoronary application of BCM or saline in a 2:1 ratio [62]. Unfortunately, the investigators did not manage to demonstrate a significant difference in adverse clinical event rate or change in LVEDVi from baseline to 6 months, assessed by an independent echocardiography core laboratory blinded to patient data. However, administration of a larger volume of BCM or alternative timing of administration could be worth further investigation. The surgical administration of injectable calcium alginate hydrogel (Algisyl), similar to BCM, has been investigated in patients with advanced heart failure [73]. A total of 78 patients with LVEF ≤ 35%, peak VO2 between 9.0 and 14.5 ml/kg/min, and a dilated LV were randomized to treatment with 15 injections or optimal medical therapy. Statistically significant improvements were observed for VO2, 6MWT distance, and NYHA functional class. There was, however, no sham procedure performed in the control group, and there were nine deaths in the Algisyl group (22.5%) vs. four deaths in the control group (10.5%), although the trial was not powered to assess mortality.

## Clinical trial design

Study endpoints of clinical trials investigating the effect of LV restoration or enhancing devices should be carefully considered. Because LV dimensions are artificially altered, they should be seen as independent variables. Parameters of LV remodeling such as LVESVi and LVEDVi have to be used to assess the efficacy of the procedure and should not be interpreted as a proxy variable for patient outcome. Preferable outcome measures to be considered for large pivotal trials are (cardiovascular) mortality, major adverse cardio- and cerebrovascular events, and (non-)heart failure hospitalizations [74]. Other (secondary) endpoints to be considered are functional capacity, severity of dyspnea on visual analogue scale (VAS) or Likert scale, and quality of life assessments. Another important consideration for pivotal randomized controlled trials investigating transcatheter restoration devices is the use of a sham procedure in the control group. A recent meta-analysis suggested that mean improvement in sham groups for percutaneous procedures can be as large as 64%, highlighting the strong placebo effects [75]. A striking example of the importance of a sham procedure is the SYMPLICITY HTN-3 sham controlled trial, which was not able to reproduce the blood pressure-lowering effects of catheter-based renal artery denervation that earlier nonsham controlled trials had observed [76]. A final consideration for device trials is that follow-up after initial results should be extended to 5 years to investigate the long-term effects.

## Future perspectives

In May 2017, new European medical device regulations (2017/745) were entered into force [77]. The new regulations increase safety and performance requirements of high-risk devices and the level of supervision of notified bodies. High-risk devices will have to be evaluated by a panel of clinical experts, devices will have to demonstrate equivalence to other safe devices in the market, and more detail will be required in clinical evaluation reports. Relevant information on medical devices will be collected in the European Database on Medical Devices (EUDAMED), and all devices will require a mandatory Unique Device Identification (UDI) so that they can be traced. There will be stricter requirements for post-market surveillance, including annual safety update reports which have to be assessed by a notified body. There is a transition period until May 2020 during which notified bodies will be designated under the new rules. Before the end of the transition period, all medical devices must be recertified under the new regulations. It will prove to be challenging for small innovative medical device manufacturers to comply with the new requirements.

Patient selection could be very important in determining which patients could have clinical benefit from implantation of a LV restoration device. As the potential benefit of LV restoration at early stages after MI is theoretically larger, early assessment of myocardial viability after MI could be a tool for risk stratification and determining the need for an early intervention. In patients with heart failure, an elementary requirement to be considered for device implantation is that medical therapy has been optimized according to heart failure guidelines, as this has been proven to promote reverse LV remodeling. A potential predictor of poor outcome could be pre-existing diastolic dysfunction, as using a device to artificially reduce the LV volume might further increase LV myocardial stiffness. The lack of consensus on the definition of diastolic dysfunction makes investigating this very challenging. A post hoc analysis of the association between pre-existing diastolic dysfunction and survival in earlier randomized controlled trials on surgical LV restoration could provide more insight.

Improvements in safety and efficacy of mechanical circulatory support devices such as left ventricular assist devices (LVAD) might reduce the need for LV restoration devices in the future. The 6-month results of the HeartMate 3 Left Ventricular Assist System (Abbott, Abbott Park, IL, USA) demonstrate a survival free of any nonsurgical bleeding, thromboembolic event, pump thrombosis, or neurological event in 69% of patients [78]. Progressive improvements could gradually move the indication for use toward earlier stages of heart failure.

## Conclusions

Both surgical and transcatheter LV restoration techniques consistently demonstrate improvements in quality-of-life measures and functional status but currently fail to demonstrate a clear survival benefit. Study designs for surgical LV restoration techniques are limited to patients that are already planned for surgery with thoracotomy, such as CABG. The noninvasive nature of transcatheter procedures allows for easier patient selection and identification of independent device-related effects. Selection of suitable study endpoints and the use of a sham control procedure is essential in clinical trial design. Transcatheter LV restoration devices show promising results in both animal and in-human studies at different stages of adverse LV remodeling after MI. The Parachute device could be beneficial in heart failure patients with a recent anterior MI, poor systolic function, and a suitable LV anatomy by reducing cardiac dimensions and end-diastolic wall stress. The PARACHUTE IV randomized controlled trial will have to demonstrate additional benefit on top of optimal medical therapy, but it is uncertain whether investigation of the device will be continued. Most of the benefit of LV restoration devices is theoretically gained at early stages after MI, using devices that can alter LV mechanical properties, such as transcatheter injection of biomaterials in the infarcted region. This will require proper selection of patients at risk of adverse LV remodeling. In the near future, it will prove to be challenging for small medical device manufacturers to comply with requirements of the 2017 European medical device regulations.
